# Quality of life outcomes associated with stigma in autistic adults and their families: a systematic review

**DOI:** 10.3389/fpsyt.2026.1911423

**Published:** 2026-09-30

**Authors:** Brian Liu, Zhonghao Zhang, Naveen Thomas, Soumitra Das

**Affiliations:** 1 The University of Melbourne, Parkville, Victoria, VIC, Australia; 2 Western Health, Melbourne, Victoria, VIC, Australia

**Keywords:** autism spectrum disorder, autistic adults, camouflaging, caregivers, family, quality of life, stigma

## Abstract

**Background:**

Autism Spectrum Disorder (ASD) is a neurodevelopmental condition characterized by impaired communication and repetitive and/or restricted behaviors or interests. Autistic adults face stigma in several forms including discrimination, negative judgement and stereotyping, which may contribute to poorer quality of life (QoL) outcomes. Whilst the impact of stigma on QoL in autistic pediatric populations have been extensively studied, autistic adults and their caregivers represent a gap in the literature.

**Aims:**

To examine different types of stigma faced by autistic adults and their family/caregivers and its impact on their quality of life, guided by World Health Organization QoL (WHOQOL) domains. which include physical health, psychological health, social relationships and environment.

**Methods:**

A systematic literature search was conducted in APA PsycInfo, Ovid MEDLINE (R) and Embase for keywords related to ASD and stigma. Studies that were eligible were peer reviewed English articles from 2013 onwards including autistic adults or their caregivers that examined at least one subtype of stigma as defined by this review. Stigma subtypes explored included internalized, public, structural, intersectional and affiliate stigma.

**Results:**

A total of 53 studies were included. Experiences of autism related stigma overwhelmingly led to poor quality of life outcomes across mental and physical health domains, relationships and in employment, social and healthcare contexts. Internalized stigma emerged after repeated exposure to experiences of public and structural stigma, resulting in reduced self-efficacy, lowered help seeking behaviors and increased pressure to mask autistic symptoms. Public stigma was largely associated with social exclusion, exacerbating feelings of shame and low self-esteem. Structural stigma, especially in healthcare and workplace environments, reinforced stigma related trauma and reduced participation and engagement. Affiliate stigma placed further strain on family and relationships, brewing resentment and alienation within families. Intersectional stigma across many marginalized identities such as gender, ethnicity and sexuality, compounded the damaging effects of autism related stigma.

**Conclusion:**

Autism related stigma acts at structural, societal and individual levels, contributing to poor QoL outcomes for autistic adults and their family/caregivers. Future studies should consolidate causal relationships between stigma and QoL, while combatting stigma at multiple levels.

## 1 Introduction

The term autism was first used by Swiss psychiatrist Eugen Bleuler in 1911 to describe a severe symptom of schizophrenia, characterized by a desire for escape from reality and to instead live in a world of hallucinations and fantasies (1). By the 1940s, as psychiatric research progressed and attitudes towards the mentally ill became more liberal, autism was redefined to coin a syndrome in children with social and behavioral differences (2). Autism spectrum disorder (ASD) is a childhood onset neurodevelopmental disorder characterized by narrow and/or repetitive patterns of behaviors, activities or interests, along with persistent deficits in social communication skills that lead to a clinically significant impairment in daily functioning across multiple contexts such as in occupational, social or educational environments (3). Underlying etiologies of ASD are a heterogenic mix of genetic and environmental risk factors, with symptoms typically first appearing by age 3 with some extending into adult life (4). The prevalence of ASD is estimated to range from 1% to 3%, representing a significant proportion of the global population (5, 6). Many autistic adults live with other comorbidities such as attention deficit hyperactive disorder (ADHD), epilepsy, anxiety and depression, which contribute to poorer overall QoL than neurotypical adults (7, 8). Stigma is thought to be a significant contributor to poorer psychosocial outcomes in autistic adults and their family or caregivers (9, 10).

Stigma is a mark of shame and social labelling stemming from negative stereotypes, which contributes to prejudiced behavior based on superficial characteristics such as disability, ethnicity or social status (11). The modern interpretation of stigma originated from psychologist Erving Goffman, outlining the “discrediting effect” of categorizing and stereotyping personal attributes based on initial appearance (12). Stigma is a dehumanizing process involving power imbalances, separation, status loss and labeled differences (13). Prior to modern understanding of ASD, it was commonly perceived through highly stigmatizing frameworks, such as attributing it to cold incompetent parenting or “emotional refrigerators” (14). In modern psychiatric literature, stigma manifests in many forms, including self-stigma, internalized stigma, affiliate stigma, public stigma, structural stigma and intersectional stigma. Self and internalized stigma are closely related; internalized stigma involves the absorption of negative stereotypes, devaluation and prejudices into self-identity, which feeds into self-stigma, whereby internalized stereotypes lead to feeling of shame and low self-esteem (15). Similarly, affiliate stigma is the internalization of stereotypes by family and caregivers of autistic individuals, exacerbating negative self-perceptions (16). Public stigma refers to damaging stereotypes that promote fear, rejection and discrimination against those with mental illness (17). Structural stigma is the systematic and institutional restriction of resources, recognition and opportunities of the stigmatized (18, 19). Intersectional stigma describes the interaction of multiple marginalized identities, exacerbating existing (20, 21).

Existing literature on ASD-related stigma and consequences is heavily focused on pediatric populations and their families, finding that autistic children had fewer friendships, greater difficulty with education and experienced a greater degree of loneliness and bullying when compared with neurotypical peers (22–25). Adult literature is relatively sparser, with some findings suggesting poorer employment opportunities and social (26, 27). However, adult populations face more complex challenges such as societal expectations of career building and family, healthcare engagement and camouflaging behaviors, adding further complexity to the experience of stigma.

Recent systematic reviews have explored stigma experienced by children with co-occurring diagnoses of ASD and intellectual disability (ID), or emphasized stigma coping strategies (9, 28). To our knowledge, there have been no comprehensive data synthesis on stigma experienced by autistic adults and their family or caregivers and its impact on quality of life within the past decade. Therefore, this review aims to elucidate types of stigma experienced by autistic adults and their families while outlining its impact on quality of life (QoL) in line with the World Health Organization QoL (WHOQOL) measure which encompasses physical health, psychological health, environment and social relationships, ultimately providing potential areas for improvement in advocacy, awareness and wellbeing (29). This measure was chosen to reflects a holistic and cross cultural approach to quality of life, facilitating further insight and priority into subjective perceptions of wellbeing. This added context is essential when examining the lived experiences of autistic adults, which is shaped by societal and structural pressures, as to not devalue their unique and complex lives into a collection of diagnostic traits.

## 2 Methods

### 2.1 Search strategy

The databases searched on Ovid in February 2026 include Ovid MEDLINE (R) ALL (2013 to February 17, 2026), APA PsycInfo (2013 to February 2026 Week 3) and Embase Classic + Embase (2013 to 2026 February 17). Additionally, controlled vocabulary terms adapted to each database were used alongside the free text terms for the autism, stigma and adult search blocks (Appendix 1). The search was conducted with the following strategy across all 3 databases:

“autis*” OR “Asperger*” OR “Pervasive Developmental Disorder*” OR “autism spectrum”

AND

“stigma*” OR “discriminat*” OR “prejudice*” OR “stereotype*” OR “negative attitude*” OR “inequalit*” OR “social exclusion”

AND

“adult*”

Additionally, the following filters were applied – Human studies, English language, 2013-current. Covidence removed duplicates automatically. Only peer reviewed articles published from 2013 onwards were considered, in line with the DSM-V incorporation of Autism, Asperger Syndrome and Pervasive Developmental Syndrome under the umbrella diagnosis of ASD and updated diagnostic criteria. This was to ensure both standardized data quality and consistency in definitions of ASD, while focusing on the most recent literature. The full search strategy can be found in **Supplementary Material**.

### 2.2 Inclusion criteria

Adults with a formal diagnosis of ASD

Self/internalized stigma, affiliate stigma, public stigma, structural stigma, intersectional stigma

English

Mixed methods, qualitative or quantitative studies

Published from 2013 onwards

The decision was made to only include formally diagnosed ASD and not self-diagnosed to provide a consistent and uniform definition of the diagnosis. Although self-diagnosis of ASD is important and increasingly valid, it is possible that these individuals may have similar diagnoses such as ADHD, Obsessive Compulsive Disorder (OCD) or other developmental diagnoses. This adds a layer of complexity which may confound interpretation of the relationship between ASD related stigma and QoL outcomes.

### 2.3 Exclusion criteria

Minors <18 years old

Papers that did not specify stigma subtype

Non-English papers

Non-human studies

Review articles, opinion articles, case studies or series, editorials, newsletters and meta-analysis

Articles with a focus on non-civilian context e.g. military

Adults with a formal diagnosis of ASD but with other comorbidities eg schizophrenia

### 2.4 Screening and data extraction

Articles searched on Ovid were imported into Covidence for title and abstract screening, full text screening and data extraction. Studies were screened and extracted by 2 independent reviewers. Study characteristics were documented in Microsoft Word (**Table 1**), including publication details such as location and authors, study design, participant demographics, type of stigma examined and main quality of life findings. Articles examining pediatric and adolescent populations were screened out manually to avoid excluding relevant studies that may have included adult populations.

**Table 1:** Summary of included studies.

Authors and publication year	Location	Study design	Study sample (age, gender and comorbidities)	Population	Assessment tool	Stigma type	Main findings
Alhusayni et al. (2025) (50)	UK	Quantitative (Experimental)	Mean Age = 23.84 +/- 5.30 n (female) = 21 n (males) = 4	Neurotypical university students (n = 25)	18 stimulus models (9 autistic, 9 non-autistic) each provided 4 silent video clips of their behavior. Questionnaire evaluated attitudes towards the models including academic success and performance, motivation to study and happiness	Public Stigma	Autistic stimulus models were rated more negatively in all academic domains and happiness compared with neurotypical individuals. Raters were blinded to the autism status of the models.
Bachmann et al. (2019) (82)	Germany	Quantitative (Cross sectional survey)	Mean Age = 31.8 +/- 11.4 n (female) = 118 n (males) = 31 n (learning disability IQ 70-84) = 26	ASD (n = 149)	Internalised Stigma of Mental Illness Scale (ISMI-10) IQ Test International standard classification of education	Self/Internalised Stigma	Higher levels of internalised stigma was associated with increasing age and lower education levels. Participants reported relatively low levels of internalised stigma, with 15.4% reporting moderate to severe levels.
Bakombo et al. (2023) (83)	Canada	Qualitative (Content analysis)	Youtube Video search n (Videos) = 59 n (Comments) = 680 n (2019 Videos) = 50 n (2019 Comments) = 500 n (2022 Videos) = 9 n (2022 Comments) = 180	Youtube videos and commenters discussing autistic people and ASD	Thematic analysis of Youtube videos and comments Sentiment analysis with Azure machine learning add in for Excel Identification of damaging language and stigmatisation	Public Stigma	Most Youtube videos were of educational purpose with comments that mostly contained negative sentiment. These comments centered around low empathy and perceptions that homogenised ASD. Use of damaging language and levels of stigma were low.
Baldwin & Costley (2015) (53)	Australia	Mixed Methods	Mean Age = 32.7 +/- 12.3 n (female) = 82	ASD adults who were a subgroup of a larger adult ASD sample (n = 82)	28 item questionnaire examining health and wellbeing, education, employment, social activities, support needs and aspirations	Public Stigma	Autistic women reported higher levels of mental health distress and lower rates of employment despite relatively high educational achievement. Barriers reported included social isolation, discrimination, bullying, unmet support needs in educational or work environments and need to mask autistic traits to prevent negative judgement.
Beck et al. (2026) (36)	USA	Qualitative (Semi-structured interviews)	Age Range = 19-68 n (female) = 7 n (male) = 7 n (non-binary) = 1	ASD (n = 15)	Semi-structured interviews examining experiences of emotional dysregulation	Public, Internalised & Structural Stigma	Most participants experienced discrimination and stigma, including in healthcare, employment and social settings, leading to disempowerment and exacerbating emotional outbursts. Lack of support and understanding paired with social rejection led to low self-worth, social masking and emotional dysregulation, causing instances of job loss, involuntary sedation and removal of care.
Belluzzo al. (2025) (37)	Italy	Qualitative (Semi structured interviews)	Age Range = 25-30 n (female) = 1	ASD (n = 1) Serious Mental Illness & Intellectual Disability (n = 12)	Semi structured interviews examining body perception, sexuality and relationships	Internalised, Public & Structural Stigma	The autistic participant reported psychosexual marginalisation underpinned by limited support services, family control and societal stigma, contributing to anxiety, shame, vulnerability to exploitation and misunderstandings about sexual identity and intimate relationships.
Black et al. (2020) (26)	Australia, Sweden, USA	Quantitative (Cross sectional survey)	Mean Age (ASD) = 36.9 +/- 12.11 n (male ASD) = 98 n (female ASD) = 137 Mean Age (Family members) = 21.2 +/- 5.7 n (male family member) = 183 n (female family member) = 47	Total (n = 687) ASD (n = 246) Family members of ASD (n = 233) Employers (n = 35) Service providers (n = 123) Researchers (n = 50)	30-point questionnaire utilising a 4-point Likert Scale on the barriers and facilitators to different stages of employment (preparing, gaining and maintaining)	Public & Structural	Employment barriers included stigma, communication difficulties, and poor understanding, with stigma driving nondisclosure. Employers underestimated communication challenges compared to autistic adults.
Bouaddi et al. (2025) (46)	Morocco	Qualitative (Semi structured interview and focus group)	Mean Age (ASD) = 23.8 +/-6.09 n (female ASD) = 2 n (male ASD) = 6 Mean Age (parents) = 50.1+/- 8.21 n (female parents) = 5 n (male parents) = 6 Mean Age (health professionals) = 33.1+/- 6.2 n (female health professionals) = 13 n (male health professionals) = 2	total (n = 40) ASD adults (n = 8) ASD caregivers (n = 11) Healthcare professionals (n = 15) Civil society representatives (n = 6)	Focus group discussions with ASD individuals to examine perceptions, experiences and challenges of autism services, healthcare and education Semi structured interviews with caregivers, health providers and civil society representatives	Public & Structural Stigma	Limited autism knowledge, services, and accommodations delayed diagnosis and support, while lifelong stigma contributed to isolation and poorer outcomes.
Brake (2025) (33)	Canada	Qualitative (Semi structured interview and focus group)	n (male) = 7 n (female) = 11 n (non-binary) = 3	ASD (n = 21)	Semi structured interviews examining autism perceptions and stigma, life and social experiences Focus group discussions	Public, Internalised and Intersectional Stigma	Autism stigma led to bullying, discrimination, internalised stigma, and camouflaging. Intersecting identities further amplified exclusion and disadvantage.
Brosnin & Gavin (2021) (84)	UK	Quantitative (Experimental)	Age Range = 18-25 n (female) = 306	Neurotypical women seeking male partner (n = 306)	Social distancing scale Rating dating profiles of autistic men based on social, physical and task attractiveness, trustworthiness and desire to date on a Likert Scale	Public & Societal Stigma	ASD stigma inversely predicted social attractiveness, task attractiveness, and dating desirability for autism-labeled profiles, directly modulating evaluative responses.
Bury et al. (2022) (85)	Australia	Quantitative (Cross sectional survey)	Mean Age = 34.89 +/-12.34 n (male) = 58 n (female) = 111 n (non-binary) = 29	ASD (n = 198)	Autism Spectrum Quotient Leach Identity Scale Autism Awareness Scale Internalised Stigma of Mental Illness Scale (ISMI) Spectrum Abilities Subscale of the Autism Spectrum Identity Scale Questionnaire on masking and sources of autism knowledge	Internalised Stigma	Learning about ASD from traditional sources such as parents, media and film were linked to higher stigma, while peer and autistic-led sources were linked to greater knowledge and stronger autistic identity.
Cage et al. (2019) (56)	UK	Quantitative (Cross sectional survey)	Mean Age = 21.44 +/-9.99 n (male) = 60 n (female) = 297 n (other) = 4	n (neurotypical participants) = 361 n (adults) = 109 n (undergraduate psychology students) = 144 n (adolescents 16-17) = 108	Vignette of autistic adults Questionnaire examining dehumanisation and autism openness, knowledge and experience	Public Stigma	Autistic individuals were dehumanised and seen as less human than neurotypical peers, although greater autism openness reduced this effect. Knowledge alone did not improve attitudes.
Caron et al. (2022) (86)	Canada & France	Quantitative (Cross sectional survey)	Mean Age (France) = 33.51 +/- 11.74 n (male) = 31 n (female) = 113 n (other) = 58 Mean Age (Quebec) = 37.03 +/-11.08 n (male) = 68 n (female) = 130 n (other) = 27	ASD Total (n = 427) French Group (n = 202) Quebec Group (n = 225)	Autism Quality of Life Measure (ASQoL) Autism Spectrum Quotient (ASQ) Questionnaire examining experiences of perceived violence	Public Stigma	French and Canadian groups reported stigma and violence, which were associated with lower quality of life across physical, mental, social, and environmental domains.
Cheng et al. (2025) (87)	UK, USA & Hong Kong	Quantitative (Cross sectional survey)	Mean Age (Hong Kong) = 22.26 +/- 4.36 n (male) = 38 n (female) = 75 n (Non-binary) = 1 n (Undisclosed) = 5 Mean Age (UK) = 28.74 +/- 10.9 n (male) = 37 n (female) = 79 n (Non-binary) = 2 n (other) = 1 n (undisclosed) = 1 Mean Age (USA) = 26.02 +/- 8.21 n (male) = 48 n (female) = 67 n (non-binary) = 3 n (transgender female) = 2 n (other) = 2	Neurotypical University Students Total (n = 368) Hong Kong Group (n = 125) UK Group (n = 120) USA Group (n = 123	Implicit Association Test (IAT) Participatory Autism Knowledge Measure (PAK-M) Culture Orientation Scale Social Distance Scale (SDS)	Public Stigma	Higher stigma was associated with lower autism knowledge, higher bias, and more individualistic values, with lower knowledge in Hong Kong than USA and UK.
Clarke et al. (2024) (73)	USA, UK, Norway	Mixed Methods	AAA n (male) = 297 n (female) = 109 EDX n (male) = 158 n (female) = 34 RI-CART n (male) = 795 n (female) = 221 MoBa n (male) = 146 n (female) = 42 SNAP n (male) = 139 n (female) = 16 QUEST n (male) = 46 n (female) = 34	Caregivers of Autistic Adults (n = 20) Adolescents and Adults with Autism Cohort (AAA) (n = 406) Early Diagnosis Cohort (EDX) (n = 192) Rhode Island Consortium of Autism Research and Treatment (RI-CART) (n = 1016) Norwegian Mother, Father, and Child Cohort (MoBa) (n = 188) Special Needs and Autism Project (SNAP) (n = 155) QUEST (n = 80)	Prevalence of Profound Autism Semi structured interviews examining quality of life of caregivers of autistic adults	Structural & Affiliate Stigma	The vast majority of caregivers reported at least one experience of perceived stigma, exacerbated by inaccurate community perceptions of autism. Caregivers also reported inadequate access to housing, healthcare and activities for their autistic adult children, further marginalising them from society.
Crompton et al. (2022) (47)	UK	Qualitative (Semi-structured interviews)	Mean Age = 24.54 +/- 4.19 n (male) = 8 n (female) = 5	ASD (n = 13)	Semi structured interviews examining attitudes and barriers to implementing autistic peer support in secondary schools Autism Quotient Score	Public & Internalised Stigma	Peer support improved belonging and reduced camouflaging, but stigma limited disclosure due to fear of judgement.
Curnow et al. (2025) (42)	UK	Qualitative (Semi-structured interviews)	Mean Age = 43.41 +/- 10.24 n (male) = 10 n (female) = 22 n (non-binary) = 2	ASD Adults Employed in Healthcare or Education Sector (n = 34)	Semi structured interview examining autistic professionals’ lived experience	Public, Structural & Internalised Stigma	ASD disclosure often led to discrimination, with communication difficulties and sensory overload increasing stress and mental health burden.
Dambrun et al. (2024) (88)	France	Quantitative (Cross sectional survey)	Mean Age = 21.0 +/- 6.0 n (male) = 126 n (female) = 342 n (other) = 19	Neurotypical Adults (n = 952)	Social Distance Scale (SDS) Feeling Thermometer Visual analog scales for social perceptions including vital forces, burden, warmth, competence and dangerousness	Public Stigma	Compared to 14 other mental illnesses, ASD was perceived favorably among French participants, in contrast to more stigmatised conditions such as schizophrenia and alcohol addiction. ASD had high vital force, competence and warmth with low burden, associated with lower stigma conditions.
Davis et al. (2024) (89)	USA	Mixed Methods	Mean Age (ASD) = 23.43 +/- 2.06 Mean Age (without ASD) = 22.35 +/-1.76	Black Emerging Adults Total (n = 34) With ASD (n = 14) Control without ASD (n = 20)	Understanding Discrimination Scale	Public & Intersectional Stigma	Black autistic adults reported intersecting racism and ableism across home, work, and social settings, cumulatively harming mental health, relationships, and employment outcomes.
Doyle et al. (2022) (76)	Global (North America, Europe, Australasia, Rest of World)	Quantitative (Cross sectional survey)	Mean Age = 38.6 +/- 10.758 n (male) = 181 n (female) = 310 n (non-binary) = 63 n (undisclosed) = 22	ASD (n = 576)	Workplace Exclusion Scale (WES) Perceived Group Inclusion Scale (PGIS)	Public, Structural & Intersectional Stigma	Race, gender and sexuality compounded existing autism related stigma. Participants who were female, non-binary, transgender or black reported reduced sense of belonging, lower authenticity and felt less supported.
Fukushima et al. (2025) (57)	Japan	Quantitative (Cross sectional survey)	n (male) = 150 n (female) = 476	Neurotypical Japanese University Students (n = 626)	Social Distance Scale (SDS) Four vignettes depicting autistic student in university classroom	Public Stigma	Disruptive behaviors and diagnosis disclosure increased public stigma, with the highest stigma when both were present. Contrastingly, those with withdrawn behaviors and closed diagnosis elicited the least stigma.
Gargallo-Nieto et al. (2025) (67)	Spain	Qualitative (Semi-structured interview)	Mean Age = 34.1 +/- 10.74 n (female) = 30	ASD (n = 30)	Semi structured interview examining ASD diagnostic process	Public, Structural & Intersectional Stigma	Late ASD diagnosis was driven by gender bias and limited adult focussed ASD services, contributing to frustration, self-blame and camouflaging, while diagnosis brought a mixture of relief and guilt or anger.
Gillions et al. (2026) (61)	UK	Qualitative (Semi-structured interviews)	Age Range = 51-73 n (female) = 15	ASD (n = 15)	Semi structured interviews examining healthcare experiences Autism Quotient Score-10 (AQ-10)	Public & Structural Stigma	Poor autism training in healthcare led to misdiagnosis, dismissal, and reduced trust, worsening mental health and promoting camouflaging, with calls for more flexible care such as telehealth or sensory accommodations.
Grahame et al. (2024) (62)	UK	Qualitative (Semi structured interviews)	Mean Age = 39.6 n (female) = 9	ASD (n = 8)	Interpretive Phenomenological Analysis (IPA)	Internalised, Public & Structural Stigma	Lack of autism-informed maternity care led to judgement, alienation, and self-doubt, while consistent support was protective.
Han et al. (2023) (34)	UK	Mixed Methods	Age Range = 18-74 n (male) = 50 n (female) = 75 n (non-binary) = 18	Total Participants (n = 144) ASD (n = 124) Parent/Caregiver of ASD (n = 38) Both Categories (n = 18)	Survey of open ended and multiple choice questions examining benefits, feasibility and barriers of stigma support program and how to best implement it	Internalised & Public Stigma	Stigma was difficult to manage alone, but disclosure commonly lead to hostility, rejection and negative responses due to a lack of knowledge surrounding ASD. Scepticism regarding ASD diagnoses perpetuated internalised feelings of low self-worth, trauma and insecurity, further contributing to masking and poor mental health.
Harada et al. (2025) (65)	Japan	Qualitative (Semi structured interviews)	Age Range = 34-46 n (male) = 3 n (female) = 4	ASD total (n = 7)	Semi structured interviews examining autistic lived experiences	Internalised, Public & Structural Stigma	Participants experienced lifelong stigma and hardship, with diagnosis bringing mixed relief and fear. Societal pressure to camouflage was linked to burnout and poorer mental health.
Hillier et al. (2020) (38)	USA	Qualitative (Focus groups)	Age Range = 20-38 n (queer) = 1 n (agender) = 1 n (nonbinary/agender) = 1 n (transgender/male) = 1	ASD who identify as LGBTQ+ (n = 4)	Questionnaire examining issues and experiences of ASD and LGBTQI+ Focus group discussions	Internalised, Public, Structural and Intersectional Stigma	Combined autism and LGBTQ+ stigma cumulatively led to alienation, identity dismissal, and reduced social connection, with poor services contributing to delayed diagnosis and reduced trust in healthcare.
Huang et al. (2022) (54)	Australia	Mixed Methods	Mean Age = 31.89 +/- 17.08 n (male) = 153 n (female) = 213 n (Nonbinary/other) = 27	ASD (n = 392)	Autism Spectrum Quotient-Short (AQ-28) Questionnaire on disclosure of ASD diagnosis	Public Stigma	ASD disclosure was often used to seek support but avoided in work and study settings due to fear of stigma and negative judgement.
Huang et al. (2023) (35)	Australia	Mixed Methods	Mean Age = 41.21 +/- 12.31 n (male) = 36 n (female) = 91 n (other) = 13 n (missing) = 3	ASD (n = 143)	Autism Spectrum Quotient Short (AQ-28) Internalised Stigma of Mental Illness Scale– Brief Version (ISMI-9) Impact of Diagnosis Scale-Revised (IODS-R)	Internalised Stigma	Most participants reported mild internalised stigma, which was linked to poorer wellbeing and masking, while diagnosis was associated with improved self-understanding and support.
Liu et al. (2024) (90)	China & Netherlands	Quantitative (Cross Sectional Survey)	Mean Age (Chinese) = 26.43 +/- 7.59 n (male) = 23 n (female) = 13 Mean Age (Dutch) = 28.00 +/- 7.35 n (male) = 18 n (female) = 11	ASD Chinese (n = 36) ASD Dutch (n = 29)	Internalised Stigma of Mental Illness-10 (ISMI-10) Survey examining life satisfaction and objective psychosocial functioning	Internalised Stigma	Chinese participants reported higher internalised stigma than Dutch participants, which was associated with poorer friendships and life satisfaction.
Mosquera et al. (2021) (39)	Spain	Qualitative (Semi structured interviews)	Mean Age = 30.2 +/-5.06 n (male) = 1 n (female) = 3 n (nonbinary) = 1	ASD (n = 5)	Photo Elicitation Interviews	Internalised & Public Stigma	Participants reported pertinent themes of rejection and need for an “endless camouflaging performance,” causing poor mental health. Although COVID-19 lockdowns reduced masking needs and facilitated more time to invest in hobbies and family, it also exacerbated autism stigma, leading to increased vulnerability, meltdowns, anxiety, depression and suicidality.
Marion et al. (2023) (55)	USA	Mixed Methods	Mean Age = 25.12 +/- 9.50 n (male) = 23 n (female) = 21 n (unreported) = 1	ASD (n = 45)	Stigma Scale Adapted Rosenberg Self-Esteem Scale Social Self-Efficacy Sub Scale Social Satisfaction Measure Adaptive Behaviour Assessment System (ABAS)	Public Stigma	Higher disclosure and discrimination stigma was linked to lower self-efficacy, self-esteem, and social satisfaction, with widespread experiences of ostracization and verbal abuse.
Marsack & Perry (2018) (70)	USA	Qualitative (Semi structured interviews)	n (female) = 46 n (male) = 5	ASD Caregivers (n = 51)	Semi structured interviews examining caregiver experiences and quality of life	Affiliate Stigma	Lack of autism knowledge led to caregiver blame and stigma, increasing isolation, relationship strain, and emotional, financial and physical burden. Caregiving for adult autistic children was often emotionally and physically exhausting, involving a lot of sacrifice and selflessness.
McLeod et al. (2019) (91)	USA	Quantitative (Cross sectional survey)	Mean Age (ASD) = 21.78 +/- 4.94 n (male) = 58 n (female) = 30 n (other) = 7	College Students Total (n = 3073) ASD (n = 95) Other Disabilities (n = 804) Neurotypical (n = 2174)	National Survey of Student Engagement National (NSSE) Longitudinal Study of Adolescent to Adult Health	Public Stigma	University students with ASD had poorer academic, social, and mental health outcomes than neurotypical peers, with few differences compared to other disability groups.
MilačićVidojević et al. (2014) (71)	Serbia	Quantitative (Cross sectional survey)	Age Range = 18-65 n (female) = 108 n (male) = 73	Neurotypical Adults (n = 181)	Family Stigma Questionnaire (FSQ) Level of Familiarity Questionnaire (LFQ) Vignettes of family/caregivers of adult ASD children	Affiliate Stigma	Stigma toward ASD families involved blame and pity, with parents more stigmatised than siblings; higher stigma was linked to older age, lower education, and low autism knowledge.
Nicolaidis et al. (2015) (63)	USA	Qualitative (Semi structured interviews)	Mean Age (ASD) = 35 n (male) = 22 n (female) = 17	ASD (n = 39) ASD Caregivers (n = 16)	Semi structured interviews focussing on healthcare experiences	Public & Structural Stigma	Healthcare stigma led to fear of disclosure and poor care, driven by stereotypes, poor communication and limited training or knowledge, with calls for better education and accommodations.
Nimante et al. (2023) (92)	Latvia	Qualitative (Semi structured interviews)	Age Range = 18-26 n (male) = 4)	ASD (n = 4)	Semi structured interviews focussing on disclosure in the workplace	Public Stigma	Participants are more likely to disclose their ASD diagnosis to close friends than colleagues or employers. Key reasons included no perceived benefit of disclosure, fear of being stigmatised, reluctance to be seen or treated differently and preference to explain support needs rather than attach autism label.
Oti-Boadi et al. (2020) (72)	Ghana	Qualitative (Semi structured interviews)	Mean Age (Parents of ASD) = 55.8 Mean Age (ASD) = 20.5	Parents of ASD (n = 9) ASD (n = 9)	Semi structured interviews examining parent experiences of caring for young adults with ASD	Affiliate Stigma	Affiliate stigma involved social exclusion and blame toward caregivers, which increased emotional burden and contributed to caregiver exhaustion, compounded by limited support in Ghana.
Pyszkowska et al. (2025) (79)	Poland	Mixed Methods	Mean Age = 27.43 +/-6.71 n (female) = 78 n (male) = 41 n (nonbinary) = 4 n (other) = 9	ASD and Queer (n = 134)	Internalized Stigma of Mental Illness Scale (ISMI) Sexual Minority Stress Scale (SMSS) Self-Stigma Scale Short (SSS-S) Collective Self-Esteem Scale (CSES) Autism Quotient-10 (AQ-10) Camouflaging Autistic Traits Questionnaire (CAT-Q) Satisfaction with Life Scale (SWLS) Open ended questions on experiences of wearing LGBTQ+ symbols	Internalised & Intersectional Stigma	ASD internalised stigma and queer minority stress were linked to lower life satisfaction and self-esteem, while stigma resistance and pride identity were protective.
Riebel et al. (2025) (48)	France	Quantitative (Cross sectional survey)	Mean Age = 36.5 +/- 10.8 n (female) = 414 n (male) = 175 n (nonbinary) = 80 n (other) = 20	ASD (n = 689)	Internalized Stigma of Mental Illness Scale -9 (ISMI-9) Depression, Anxiety and Stress Scale (DASS) Self Compassion Scale Externalized and Internalized Shame Scale (EISS) Camouflaging Autistic Traits Questionnaire (CAT-Q)	Internalised Stigma	A sizeable proportion of participants (45.2%) reported moderate to elevated self-stigma, which was correlated to higher rates of depression, mediated by internalised feelings of shame. Self-compassion was protective as it reduced the strength of this relationship, while camouflaging had no effect.
Sagar et al. (2024) (40)	India	Qualitative (Semi structured interviews)	Age Range = 21-50 n (male) = 10 n (female) = 10	ASD (n = 20)	Semi structured interview and focus groups examining stimming behaviors in the workplace	Internalised & Public Stigma	Autistic employees felt pressure to suppress stimming behaviors due to fear of mockery or being perceived as unprofessional. Suppressing stimming increased stress, anxiety and reduced productivity. Stimming behaviors was a protective behavior that supported emotional regulation and stress relief.
Soeker (2020) (52)	South Africa	Qualitative (Semi structured interviews)	Age Range = 21-53 n (male) = 7 n (female) = 3	ASD (n = 10)	Semi structured interviews on transitioning into the workplace from skills training programs	Public Stigma	Workplace stigma toward autistic behaviors, including stimming and emotional dysregulation, hindered labor market transition and was linked to increased anxiety and distress.
Soffer & ArgamanDanos (2021) (93)	Israel	Quantitative (Cross sectional survey)	Mean Age = 28.4 +/- 6.9 n(male) = 51 n (female) = 35	ASD (n = 89)	Perceived Devaluation-Discrimination Scale Rosenberg’s Self Esteem Scale	Public Stigma	Higher levels of perceived public stigma were linked to lower self-esteem, despite being low in this study. Self-labelling of Asperger’s Syndrome versus ASD was not significantly linked to perceived public stigma or self-esteem.
Stein Duker et al. (2022) (64)	USA	Qualitative (Semi structured interviews)	n (male) = 3 n (female) = 29	ASD Caregivers (n = 31)	Ritvo Autism Asperger diagnostic Scale-Revised (RAADS-R) Semi structured interviews examining caregiver perspectives on primary healthcare	Public & Structural Stigma	Caregivers reported fragmented, inconsistent healthcare for autistic adults, with provider stigma and misinterpretation of autistic behaviors limiting access and quality of care. Better care was suggested to be more accommodating, flexible and considerate of neurodivergence.
Tamura et al. (2025) (31)	Japan	Quantitative (Cross sectional survey)	Mean Age = 37.5 +/- 9.8 n (male) = 146 n (female) = 120 n (nonbinary) = 14 n (other) = 5 n (undisclosed) = 2	ASD = 287	Autism Spectrum Quotient 10-item short version (AQ-J10) Patient Health Questionnaire-9 (PHQ-9) Generalized Anxiety Disorder-7 (GAD-7) Liebowitz Social Anxiety Scale (LSAS) Camouflaging Autistic Traits Questionnaire–Japanese version Warwick–Edinburgh Mental Well-Being Scale (WEMWBS) Stigma Consciousness Scale Individualistic Strategy Use Collective Strategy Use	Public Stigma	Higher level of perceived stigma was associated with greater need to camouflage autistic traits. Camouflaging mediated the relationship between stigma and mental health outcomes including generalised anxiety, social anxiety and depression, but not mental wellbeing.
Tan et al. (2024) (94)	Australia	Qualitative (Semi structured interviews)	Mean Age = 36.3 +/- 9.1 n (female) = 13 n (male) = 3 n (nonbinary) = 5	ASD (n = 21)	Semi structured interviews examining experiences of stigma at Australian universities	Public & Structural Stigma	ASD stigma culminated in microaggressions and labels of being “retarded” or “exotic.” University campuses were seen as inaccessible and bureaucratic, which were hostile to students with disability due to a lack of adjustments. Students felt compelled to self-advocate, which contributed to increased attrition and delayed careers.
Treweek et al. (2019) (51)	UK	Qualitative (Semi structured interviews)	Mean Age = 37.33 +/- 15.02 n (male) = 8 n (female) = 4	ASD (n = 12)	Semi structured interviews examining stereotypes of autistic people	Public Stigma	Participants reported a chief stereotype that autistic people are “weird,” resulting in experiences of social exclusion, bullying and loss of individuality and identity.
Underhill et al. (2024) (41)	USA	Qualitative (Semi structured interviews)	Mean Age = 20.70 +/- 2.31 n (male) = 9 n (female) = 1	ASD College Students (n = 10)	Semi structured interviews examining experiences and management of peer stigma	Internalised & Public Stigma	Concealing autism was linked to anxiety, poorer academic performance, and worse mental and physical health, while internalised stigma reduced belonging, access to support, and meaningful social relationships.
Wallisch et al. (2023) (80)	USA	Quantitative (Cross sectional survey)	Mean Age (ASD) = 32.19 +/- 10.80 n (male) = 39 n (female) = 19 Mean Age (ASD + LGBTQ+) = 32.08 +/- 8.58 n (male) = 9 n (female) = 27 n (other) = 26	ASD Total (n = 120) ASD Only (n = 58) ASD + LGBTQ+ (n = 62)	2019 National Survey on Health and Disability (NSHD) survey questions on health and unmet healthcare needs	Public, Structural & Intersectional Stigma	LGBTQI+ autistic adults had poorer physical and mental health and more unmet healthcare needs and comorbidities than straight autistic adults, with anti-discrimination laws linked to reduced unmet needs.
Ward et al. (2026) (95)	UK	Qualitative (Semi structured interview)	Age Range (ASD) = 35-65 n (male) = 1 n (female) = 5 Family Members n (female) = 6	ASD (n = 6) ASD Family Members (n = 6)	Semi structured interviews examining phenomenology of later life diagnosis of ASD	Public stigma	Autism stereotypes led to workplace dismissal and nondisclosure, while delayed diagnosis increased emotional distress and reduced support. Earlier diagnosis was seen as beneficial.
Whelpley & May (2023) (58)	USA	Quantitative (Experimental)	Interviewees Age Range = 18-25 ASD Group n (male) = 6 n (female) = 9 Neurotypical Group n (male) = 4 n (female) = 11 Raters Age Range = 18-25 n (female) = 248 n (male) = 66 n (nonbinary) = 2	Interviewees ASD College Students (n = 15) Neurotypical College Students (n = 15) Raters Neurotypical College Students (n = 314)	Mock interviews of 15 ASD and 15 neurotypical college students rated by neurotypical college students on measures of overall impression, performance and hireability	Public Stigma	Autistic candidates were rated less favorably in areas of likeability, confidence and trustworthiness and less hireable than equally qualified neurotypical peers. This trend was reversed in the absence of social and visual cues.
Yoon et al. (2024) (66)	South Korea	Qualitative (Semi structured interviews)	Mean Age = 30.29 +/- 6.27 n (female) = 4 n (male) = 10	ASD (n = 14)	Semi structured interviews examining secondary school experiences of autism related discrimination	Public & Structural Stigma	Korean autistic adults reported bullying, victim-blaming, and systemic ableism, leading to long-term mental distress, nightmares, trauma, exclusion, and poorer education and employment outcomes. This was exacerbated by a culture of elitism, ableism and authoritarianism.
Zhuang et al. (2025) (30)	Australia, Global	Quantitative (Cross sectional survey)	Mean Age = 35.5 +/- 11.8 n (female) = 137 n (male) = 64 n (other) = 21 n (undisclosed) = 3	ASD (n = 225)	Camouflaging Autistic Traits Questionnaire (CAT-Q) Broad Autism Type Questionnaire (BAPQ) Brief Fear of Negative EvaluationII Scale (BFNE-II) Rosenberg Self-Esteem Scale (RSES) Multicomponent In-group Identification Scale (MIIS) Perceived Stigma Measure (PSM) Vulnerability Experiences Quotient (VEQ) Tightness Looseness Scale (TLS) Self Construal Scale (SCS)	Public Stigma	Greater camouflaging was linked to lower self-esteem and higher perceived stigma, with fear of negative peer evaluation acting as the main mediator.

### 2.5 Risk of bias

Risk of bias was assessed by two independent reviewers using the JBI critical appraisal tools (cross-sectional studies, quasi-experimental studies, or qualitative studies) or the Mixed Methods Appraisal Tool (MMAT) depending on the study design. A third reviewer resolved conflicts. Studies had a risk of bias that ranged from low to moderate.

## 3 Results

A total of 2459 studies were identified in the initial search strategy, detailed in a PRISMA Flow Diagram (**Figure 1**). A total of 2313 studies were excluded in the initial abstract screening stage as these studies did not meet inclusion criteria, for reasons such as pediatric populations, lack of quality of life outcomes reported and no exploration of stigma. Following abstract screening, 146 full-text articles were assessed for eligibility, with 53 articles ultimately included in this review. Reasons of exclusion during the full text screening stage included studies that incorporated a large proportion of self-diagnosed ASD, populations with multiple confounding comorbidities such as ADHD and OCD, a lack of stigma as the central exposure or insufficient discussion of quality of life outcomes. Of the articles included, there were 8 mixed methods studies, 17 cross-sectional, 25 qualitative, 3 quantitative (**Table 1**). In terms of location, 5 were conducted in Australia, 7 were multi-country, 11 from USA, 10 from UK, 3 from Japan, 2 from Canada, 2 from Spain, 2 from France, 1 from Ghana, 1 from Latvia, 1 from South Africa, 1 from South Korea, 1 from India, 1 from Poland, 1 from Morocco, 1 from Germany, 1 from Italy, 1 from Serbia and 1 from Israel. The number of participants in each study ranged from 1 to 3073.

**Figure 1:**
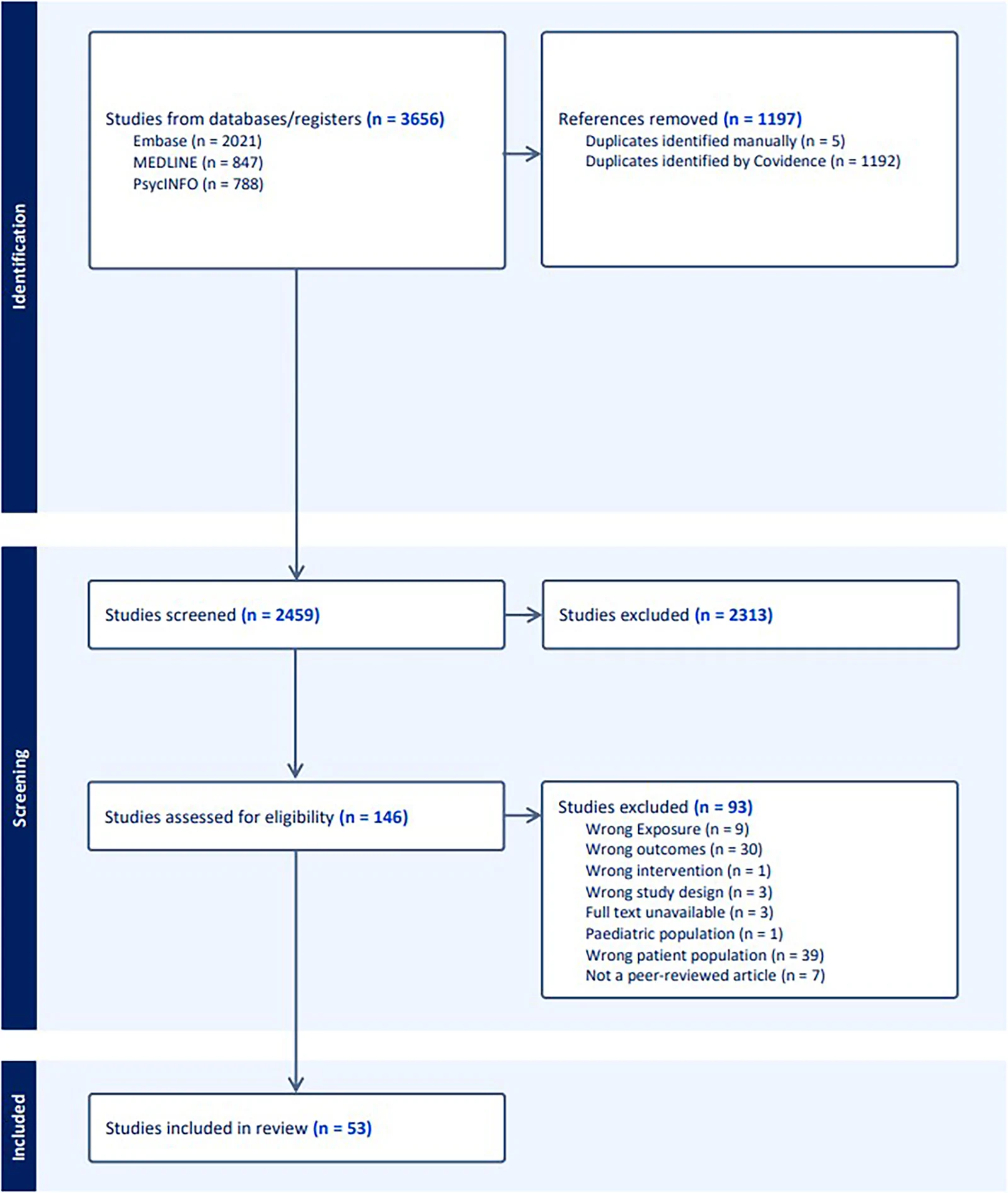
PRISMA Flow Diagram.

Articles were categorized based on the type of stigma they investigated based on definitions outlined in the introduction, including internalized and/or self-stigma, affiliate stigma, public stigma, structural stigma and intersectional stigma. Quality of life domains were centered around WHOQOL guidelines, including physical health, psychological health, social relationships and environment, and were discussed within the stigma subcategories if present.

## 4 Discussion

This review synthesized findings from 53 studies, finding that the vast majority of autistic adults experience stigma throughout their life in differing environments including in education, healthcare, employment, community and at home (**Table 1**). Experiences of autism related stigma overwhelmingly lead to poorer outcomes across the World Health Organization QoL domains. Camouflaging emerged as a key mediator between experiences of stigma and lower quality of life (30, 31). Camouflaging or masking is the deliberate concealment of autistic behaviors and mannerism due to fear of judgement or social repercussions, which is both mentally and physically exhausting (32). Additional mediators included reduced help-seeking behavior and reluctance to disclose diagnosis.

### 4.1 Internalized stigma

Internalized stigma was a consistently represented stigma subtype, explored by 18/53 studies (33.96%).

Internalized stigma was characterized by the self-adoption of deficit-based perceptions of autism, resulting in reduced self-esteem, shame and pressure to camouflage (33–35). Internalization of negative beliefs was frequently described as a downstream consequence of repeated experiences of public and structural stigma, suggestive of how those stigma subtypes may be foundational causative agents of other forms of stigma (36–38).

Camouflaging emerged as a key mediator between internalized stigma and poorer quality of life outcomes (39–41). Whilst these behaviors may be beneficial in the short term in avoiding social exclusion and prejudice, it has detrimental effects on mental health in the long term, including emotional burnout and lowered self-esteem (33, 42). This negative impact is likely because it forces autistic individuals to be guarded and suspicious of their peers, enacting a heavy mental toll and contributing to exhaustion. These behaviors are also closely associated with elevated levels of anxiety, depression and diminished sense of wellbeing in the wider literature, reinforcing camouflaging as a key driver of poor mental health (43–45).

Internalized stigma also contributed to reduced sense of agency and help-seeking behavior, likely arising from feelings of inadequacy and hopelessness (41, 46). This deficit may reinforce the deficit promoting mechanisms of public and structural stigma, as affected individuals are less likely to advocate for their needs or seek assistance in the face of despondency. Therefore, as autistic individuals become more segregated from community and support networks, cycles of disadvantage are perpetuated.

Ultimately, these findings suggest public and structural stigma both facilitate the emergence of internalized stigma. Thus, there is a need for greater exploration of protective factors such as a strong autistic identity, autism peer support groups, self-compassion and understanding, which may alleviate the pressure to camouflage and allow greater freedom to express oneself freely (47, 48). Support programs such as Standing Up For Myself (STORM) have shown promise in unpacking emotional hardships, engaging in peer support and improving self-advocacy in adults with intellectual disability, with potential to be adapted to autistic populations (49).

### 4.2 Public stigma

Public stigma was by far the most explored stigma subtype, covered in 43/53 studies (81.13%), emerging through stereotypes, discrimination and exclusionary practices. This is suggestive of its high visibility and easier measurability compared to other stigma subtypes, but should be interpreted cautiously as evidence of greater importance as other stigma subtypes may be less consciously recognized and readily measured.

Public stigma was characterized by deficit-based judgements, including those of social, workplace and academic incompetence. This was in addition to assumptions of impaired capacity for intimate relationships and stereotypes of eccentricity and awkwardness (50–52). These assumptions perpetuate negative stereotypes in the public consciousness, leading to social rejection, job loss and dismissal by healthcare professionals. Additionally, public stigma frequently emerged in subtle forms of exclusion rather than explicit hostility. Autistic adults reported frequent experiences of patronizing treatment, infantilization and microaggressions (36, 53). These hidden forms of stigma may be especially damaging as they are often ambiguous and therefore difficult to challenge, which can be easily overlooked. Repeated experiences of invalidation may gradually undermine self-esteem and confidence, further deteriorating confidence and agency.

Public stigma commonly lead to withholding autism diagnoses in professional settings due to the feared anticipation of discrimination, gossiping and unfair treatment (26, 42, 54, 55). The potential benefits of disclosure, such as greater authenticity, peer understanding and room for flexible accommodations, is often outweighed against risks such as job loss, altered treatment and inhibited career growth. This balancing act highlights a constant struggle in threatening environments that promote suspicion and guardedness rather than comfort. This represents a significant psychosocial burden which may further contribute to camouflaging, as anticipated prejudice fuels anxiety and further restricts workplace participation, freedom of self-expression and engagement.

Studies examining neurotypical attitudes towards autistic individuals found that the autistic cohorts were overwhelmingly rated more negatively in academic performance, happiness, social distancing and hireability, while being considered less human than neurotypical adults (50, 56–58). Interestingly however, in the absence of visual and social cues such as behavior and mannerisms, this trend reversed as autistic job interview candidates were rated more favorably than their neurotypical counterparts (58). This suggests that public stigma stems from surface level social signaling rather than actual competence or knowledge. Thus, autism-related differences in communication and behavior are misinterpreted as incompetence, inadequacy and poor communication.

There is a significant gap in the literature for public stigma reduction interventions, as available studies overemphasize white pediatric populations, while over relying on online educational formats that have a high risk of bias (59) Such interventions should lead and co-designed by autistic individuals with experience and knowledge of autism-related stigma, combining both educational approaches and opportunities to interact with autistic people to dispel stereotypes and dehumanizing attitudes. One such approach is the Beyond Spectrums theater production, which engages the audience with autistic narratives and reduce stigmatizing beliefs through empathy and understanding (60). Ultimately, attempts to combat public stigma should aim to emphasize autistic strengths and diversity and promote nuanced perceptions of ASD that moves towards a strengths-based understanding (34).

### 4.3 Structural stigma

Structural stigma was explored in 17/53 (32.08%) studies and was found to be deeply embedded across government policy, practices and institutions across healthcare, employment and educational contexts.

Studies consistently suggested that autistic adults faced healthcare discrimination, including experiences of dismissal, judgement and downplaying of symptoms by healthcare professionals leading to delayed or missed diagnosis, reduced help-seeking behavior and causing disempowerment (36, 46, 61–64). These experiences exacerbate internalized feelings of low self-worth, identity insecurity and self-doubt, stemming from a lack of appropriate autism education and training for healthcare professionals. Systemically, current healthcare institutions are ill-equipped to recognize and accommodate the additional needs of autistic adults such as sensory adaptations and consistent support staff with ASD knowledge. Thus, healthcare institutions require reform to implement ASD related training and education, adaptive practices to accommodate the diverse presentations of autism, and expansion of autism support services.

Institutional norms in employment and educational contexts were suggested to privilege neurotypical modes of communication, social interaction and productivity while disadvantaging autistic adults. Exclusionary workplace politics, hierarchies and policies, alongside a lack of adaptive practices to support autistic individuals promoted camouflaging behaviors and reduced willingness to disclose diagnosis due to fear of provoking further discrimination (26, 42, 65, 66). Communication was commonly identified as the most challenging barrier to employment for autistic candidates, while employers inversely rated it as the least challenging, highlighting a disparity in employer understanding of autistic employee needs (26). These findings highlight a desperate need for greater flexibility to accommodate the needs of autistic employees while ensuring safe spaces for disclosure.

Structural stigma may also exacerbate internalized and public stigma by reinforcing deficit-based narratives and normalizing exclusionary practices (61, 62, 65). This aligns with the literature that suggest institutionally embedded forms of discrimination plays a major role in shaping prejudiced public attitudes and thereby individual experiences of prejudice (18). Several studies have highlighted that repeated experiences of structural invalidation may lead to diminished self-efficacy, loss of confidence in support services and increased mental distress (46, 61, 67).

Stigma reduction interventions at systemic levels are required, such as autism informed policies, supportive disclosure environments, accommodations such as sensory adaptations or flexible appointments, alongside further staff education and training in how to manage (68, 69).

### 4.4 Affiliate stigma

A total of 4/53 studies (7.55%) explored affiliate stigma, examining cohorts across USA, UK, Norway, Ghana and Serbia. It was comparatively understudied compared to other stigma subtypes, representing a gap in the literature as it overlooks family and caregivers as potential amplifiers or secondary recipients of autism stigma.

Affiliate stigma was found to stem primarily from a place of misunderstanding and lack of knowledge about ASD, leading to negative assumptions about poor parenting being the cause of disruptive autistic behaviors rather than neurodevelopmental in nature (70–72). In support of this, parents of autistic adults were significantly more stigmatized than siblings, likely due to a false attribution of responsibility for causing ASD (71). Furthermore, these perceptions are exacerbated by the invisible nature of ASD, with socially inappropriate behaviors seen as a reflection of caregiver competence and the individual, reinforcing labels and stereotypes such as “fanatic”, “goofy” and “parents from hell” (70).

Affiliate stigma was consistently associated with negative judgement and social exclusion from friends, family and community, fed by false assumptions of incompetence and patterns of blame (70, 72). Experiences of social judgement may limit autonomy and independence as fear of discrimination minimizes social engagement, reducing access to protective factors such as support networks and healthcare. Alienation is perpetuated by the complex needs of autistic adult children, such management of sensory overload in the home environment, the need for constant supervision and lack of privacy, which brews resentment, exhaustion and missed social opportunities (70). These findings are suggestive of the inherently isolating nature of caregiving for autistic adults by positioning social participation and competent caregiving as potentially mutually exclusive.

Both social isolation and caregiver burnout cumulatively contribute to psychological exhaustion and distress. Many parents were unable to work due to caregiving commitments, resulting financial difficulties and anxiety regarding the uncertainty of their autistic adult child’s futures (72). These findings suggest that family and caregivers of autistic adult children require additional support services to promote wellbeing and prevent career burnout. However, parents reported significant difficulty in accessing respite care, housing and healthcare for their autistic adult children, due to lack of appropriate training and knowledge surrounding ASD, alongside scarce available services to begin with (73).

Addressing affiliate stigma will require increased support caregivers in the form of wider respite services, opportunities for peer support and psychoeducation for parents and careers of autistic adults. Although the literature is sparse, a proof-of-concept psychosocial stigma support program (SOLACE) improved mental health among parents of autistic children, utilizing a combination of online peer support sessions, stigma resistance and coping strategy education (74).

### 4.5 Intersectional stigma

Intersectional stigma was discussed in 7/53 studies (13.21%), exploring the convergence of ASD with other marginalized identities including gender, race and sexuality. These studies consistently suggested that additional marginalized identities tend to compound and exacerbate experiences of autism related stigma (33, 38). This supports an intersectional approach to stigma that incorporates the cumulative effects of different marginalized identity into consideration (75).

One study found that autistic adult employees who identified as non-binary, female or as a racial minority, reported reduced workplace engagement and sense of belonging compared with autistic employees who identified as white and male (76). This finding suggests that low levels of workplace satisfaction and fulfillment may be correlated with additional marginalized identities (76). Currently, white males from high socioeconomic cohorts are privileged in access to support services and diagnostic pathways, particularly within Western nations, indicative of institutional policies that inherently handicap minorities (67, 76). This bias reflects a broader consensus in the literature, suggesting that minorities are commonly rendered invisible in government policymaking and service delivery (77, 78). In autistic adults, this results in fragmentation of identity and greater barriers in accessing healthcare services or achieving career success, thereby prolonging suffering and hardship.

Additionally, intersectional stigma may pressure individuals to conform to the status quo of neurotypical behaviors and thereby worsen internalized stigma, further contributing to self-doubt (79, 80). Such pressures were closely tied to reduced workplace inclusion and authenticity, reduced life satisfaction and lowered self-esteem, likely due to a greater need to camouflage and act guardedly (76, 79).

Intersectional stigma reduction interventions should therefore utilize intersectionality informed strategies, such as incorporating lived experience and guidance from autistic underrepresented identities (20, 81).

### 4.6 Limitations

There were several limitations within this review. Only three databases (Ovid MEDLINE (R), Embase and APA PsycArticles) were searched, which may have inadvertently excluded relevant studies in other databases. Additionally, studies demonstrated inconsistencies in stigma terminology, thus limiting the comparability of studies and increasing the risk of conceptual overlap between stigma subtypes. Synthesis of evidence was also complicated by bias in categorizing studies within this review’s stigma subtype definitions. Further, as most studies were qualitative or cross-sectional in design, definitive causal and temporal relationships could not be made between stigma and reported quality of life outcomes. This was further complicated by a lack of consistent and standardized measures of QoL outcomes, thereby necessitating inferences of the relationship between stigma exposure and QoL. Studies also displayed a bias towards Western populations with a minority of underrepresented cultures, limiting the generalizability of these findings to other cultural contexts.

### 4.7 Conclusion

This review aimed to synthesize evidence exploring the relationship between stigma and QoL domains in autistic adults and their caregivers. Qualitative and quantitative studies offered a snapshot into internalized stigma, structural stigma and public stigma, with a clear gap in affiliate and intersectional stigma. QoL outcomes, albeit discussed, were more difficult to explore due to absence of direct measurements of stigma and associated QoL outcomes. Stigma was found to operate across structural, societal and personal pathways, ultimately having detrimental impacts on mental health, relationships, social participation, employment and healthcare access.

Future research should adopt longitudinal study designs to determine causal pathways between stigma experience and quality of life outcomes. More psychometrically validated tools to measure autism related stigma subtypes should be developed, especially for difficult to measure stigma subtypes such as structural stigma. Stigma reduction strategies must ultimately incorporate elements of structural reform, public education and individual level support to address the full spectrum of autism related stigma.

## Controlled vocabulary terms

Medline (MeSH): “child development disorders, pervasive” OR “autism spectrum disorder” OR “asperger syndrome” OR “autistic disorder” AND “Social Stigma” AND Adult” OR “Young “Adult”

APA Psycinfo (APA Thesaurus): “autism spectrum disorders” OR “autistic traits” OR “autism spectrum disorders (attitudes toward)” OR “neurodiversity” AND “stigma” OR “mental health stigma” OR “self-stigma” OR “Social Exclusion” AND “Middle Adulthood” OR “Emerging Adulthood” OR “Older Adulthood”

Embase (Emtree): “autism” OR “asperger syndrome” OR “pervasive developmental disorder not otherwise specified” AND “social stigma” OR “stigma” OR “self stigma” OR “social exclusion” AND “young adult” OR “adult”

## References

[1] Evans B . How autism became autism: The radical transformation of a central concept of child development in Britain. Hist Hum Sci. (2013) 26:3–31. doi: 10.1177/0952695113484320 24014081 PMC3757918

[2] Kanner L . Autistic disturbance of affective contact. Nervous Child. (1943) 2:217–50.

[3] American Psychiatric Association . Diagnostic and Statistical Manual of Mental Disorders, 5th ed. (Washington, DC 20024, American Psychiatric Association Publishing, Inc.) (2013). doi: 10.1176/appi.books.9780890425596.

[4] Sauer AK Stanton JE Hans S Grabrucker AM . Autism spectrum disorders: Etiology and pathology. In: Grabrucker AM , editor. Autism Spectrum Disorders. Exon Publications Copyright: The Authors, Brisbane (AU) (2021). 34495619

[5] Australian Bureau of Statistics . Autism in Australia, 2022. ABS, Belconnen ACT 2616 (2024). Available online at: https://www.abs.gov.au/articles/autism-australia-2022 (Accessed April 28, 2026).

[6] Zeidan J Fombonne E Scorah J Ibrahim A Durkin MS Saxena S . Global prevalence of autism: A systematic review update. Autism Res. (2022) 15:778–90. doi: 10.1002/aur.2696 35238171 PMC9310578

[7] Lord C Brugha TS Charman T Cusack J Dumas G Frazier T . Autism spectrum disorder. Nat Rev Dis Primers. (2020) 6:5. doi: 10.1016/s0140-6736(18)31129-2 31949163 PMC8900942

[8] Ayres M Parr JR Rodgers J Mason D Avery L Flynn D . A systematic review of quality of life of adults on the autism spectrum. Autism. (2018) 22:774–83. doi: 10.1177/1362361317714988 28805071

[9] Han E Scior K Avramides K Crane L . A systematic review on autistic people's experiences of stigma and coping strategies. Autism Res. (2022) 15:12–26. doi: 10.1002/aur.2652 34881514

[10] Papadopoulos C Lodder A Constantinou G Randhawa G . Systematic review of the relationship between autism stigma and informal caregiver mental health. J Autism Dev Disord. (2019) 49:1665–85. doi: 10.1007/978-1-4614-6435-8_102321-1 PMC6450836 30569408

[11] Pescosolido BA Martin JK . The stigma complex. Annu Rev Sociol. (2015) 41:87–116. doi: 10.1146/annurev-soc-071312-145702 26855471 PMC4737963

[12] Goffman E . Stigma: Notes on the Management of Spoiled Identity. Simon and Schuster, Willoughby North, NSW 2068 (2009).

[13] Link BG Phelan JC . Conceptualizing stigma. Annu Rev Sociology. (2001) 27:363–85. doi: 10.1146/annurev.soc.27.1.36341139587

[14] Kanner L . Problems of nosology and psychodynamics of early infantile autism*. Am J Orthopsychiatry. (1949) 19:416–26. doi: 10.1111/j.1939-0025.1949.tb05441.x 18146742

[15] Fernández D Grandón P López-Angulo Y Vladimir-Vielma A Peñate W Díaz-Pérez G . Internalized stigma and self-stigma in people diagnosed with a mental disorder. One concept or two? A scoping review. Int J Soc Psychiatry. (2023) 69:1869–81. doi: 10.1177/00207640231196749 37646436

[16] Mak WWS Cheung RYM . Affiliate stigma among caregivers of people with intellectual disability or mental illness. J Appl Res Intellectual Disabil. (2008) 21:532–45. doi: 10.1111/j.1468-3148.2008.00426.x40046247

[17] Parcesepe AM Cabassa LJ . Public stigma of mental illness in the United States: A systematic literature review. Adm Policy Ment Health. (2013) 40:384–99. doi: 10.1007/s10488-012-0430-z 22833051 PMC3835659

[18] Hatzenbuehler ML Link BG . Introduction to the special issue on structural stigma and health. Soc Sci Med. (2014) 103:1–6. doi: 10.1016/j.socscimed.2013.12.017 24445152

[19] Hatzenbuehler ML . Structural stigma: Research evidence and implications for psychological science. Am Psychol. (2016) 71:742–51. doi: 10.1037/amp0000068 27977256 PMC5172391

[20] Turan JM Elafros MA Logie CH Banik S Turan B Crockett KB . Challenges and opportunities in examining and addressing intersectional stigma and health. BMC Med. (2019) 17:7. doi: 10.1186/s12916-018-1246-9 30764816 PMC6376691

[21] Jackson-Best F Edwards N . Stigma and intersectionality: A systematic review of systematic reviews across HIV/AIDS, mental illness, and physical disability. BMC Public Health. (2018) 18:919. doi: 10.1186/s12889-018-5861-3 30049270 PMC6062983

[22] Bauminger N Kasari C . Loneliness and friendship in high-functioning children with autism. Child Dev. (2000) 71:447–56. doi: 10.1111/1467-8624.00156 10834476

[23] Petrina N Carter M Stephenson J . The nature of friendship in children with autism spectrum disorders: A systematic review. Res Autism Spectr Disord. (2014) 8:111–26. doi: 10.1016/j.rasd.2013.10.01642574925

[24] Rowley E Chandler S Baird G Simonoff E Pickles A Loucas T . The experience of friendship, victimization and bullying in children with an autism spectrum disorder: Associations with child characteristics and school placement. Res Autism Spectr Disord. (2012) 6:1126–34. doi: 10.1016/j.rasd.2012.03.00442574925

[25] Brede J Remington A Kenny L Warren K Pellicano E . Excluded from school: Autistic students’ experiences of school exclusion and subsequent re-integration into school. Autism Dev Lang Impairments. (2017) 2:2396941517737511. doi: 10.1177/2396941517737511

[26] Black MH Mahdi S Milbourn B Scott M Gerber A Esposito C . Multi-informant international perspectives on the facilitators and barriers to employment for autistic adults. Autism Res. (2020) 13:1195–214. doi: 10.1002/aur.2288 32170919

[27] Howlin P Goode S Hutton J Rutter M . Adult outcome for children with autism. J Child Psychol Psychiatry. (2004) 45:212–29. doi: 10.1111/j.1469-7610.2004.00215.x 14982237

[28] Meulien C Baghdadli A . A systematic review of the stigma experienced by people with autism spectrum disorder associated with intellectual disabilities and by their family caregivers. J Autism Dev Disord. (2025) 55:3955–72. doi: 10.1007/s10803-024-06435-x 39052202

[29] World Health Organization . The World Health Organization quality of life assessment (WHOQOL): Position paper from the World Health Organization. Soc Sci Med. (1995) 41:1403–9. doi: 10.1016/0277-9536(95)00112-k 8560308

[30] Zhuang S Bougoure M Leong D-J Dean L Reddrop S Naragon-Gainey K . Examining an integrated path model of psychological and sociocultural predictors of camouflaging in autistic adults. Autism. (2025) 29:2328–43. doi: 10.1177/13623613241262110 39066620 PMC12332224

[31] Tamura M Cage E Perry E Hongo M Takahashi T Seto M . Understanding camouflaging, stigma, and mental health for autistic people in Japan. Autism Adulthood. (2025) 7:52–65. doi: 10.21203/rs.3.rs-2614748/v2 40151661 PMC11937760

[32] Perry E Mandy W Hull L Cage E . Understanding camouflaging as a response to autism-related stigma: A social identity theory approach. J Autism Dev Disord. (2022) 52:800–10. doi: 10.1007/s10803-021-04987-w 33788076 PMC8813820

[33] Brake J . Between gaining acceptance and avoiding harm: Navigating stigma and its consequences among autistic individuals. Disability Soc. (2025) 40:1714–39. doi: 10.1080/09687599.2024.237449137339054

[34] Han E Scior K Heath E Umagami K Crane L . Development of stigma-related support for autistic adults: Insights from the autism community. Autism. (2023) 27:1676–89. doi: 10.1177/13623613221143590 36597933 PMC10374993

[35] Huang Y Trollor JN Foley K-R Arnold SRC . I've spent my whole life striving to be normal”: Internalized stigma and perceived impact of diagnosis in autistic adults. Autism Adulthood. (2023) 5:423–36. doi: 10.1089/aut.2022.0066 38116050 PMC10726184

[36] Beck KB MacKenzie KT Kumar T Breitenfeldt KE Chang JC Conner CM . The world’s really not set up for the neurodivergent person”: Understanding emotion dysregulation from the perspective of autistic adults. Autism Adulthood. (2026) 8:68–78. doi: 10.1089/aut.2023.0214 41048403 PMC12490785

[37] Belluzzo M Giaquinto V Volpe D Esposito C De Alfieri E Amodeo AL . Navigating body perception, affectivity, intimacy, gender identity, and sexuality: An exploratory qualitative study in young adults with SMI, IDs, and ASD in a community setting. Int J Environ Res Public Health. (2025) 22:722. doi: 10.3390/ijerph22050722 40427837 PMC12110811

[38] Hillier A Gallop N Mendes E Tellez D Buckingham A Nizami A . LGBTQ + and autism spectrum disorder: Experiences and challenges. Int J Transgend Health. (2020) 21:98–110. doi: 10.1080/15532739.2019.1594484 33005905 PMC7430467

[39] Lois Mosquera M Mandy W Pavlopoulou G Dimitriou D . Autistic adults’ personal experiences of navigating a social world prior to and during Covid-19 lockdown in Spain. Res Dev Disabil. (2021) 117:104057. doi: 10.1016/j.ridd.2021.104057 34371305 PMC8413264

[40] Sagar E Khera SN Garg N . I wish they'd just let us be.” Experiences of Indian autistic individuals around stimming behaviors at the workplace. Autism Adulthood. (2024) 6:474–84. doi: 10.1089/aut.2022.0096 40018063 PMC11861062

[41] Underhill JC Clark J Hansen RS Adams H . Exploring autistic college students’ perceptions and management of peer stigma: An interpretative phenomenological analysis. J Autism Dev Disord. (2024) 54:1130–42. doi: 10.1007/s10803-022-05867-7 36547792

[42] Curnow E Maciver D Johnston L Murray M Johnstone-Cooke V Utley I . Learning from the experiences of autistic professionals working in health and education. Autism Adulthood. (2025). doi: 10.1089/aut.2024.0168 42383264 PMC13315297

[43] Beck JS Lundwall RA Gabrielsen T Cox JC South M . Looking good but feeling bad: "Camouflaging" behaviors and mental health in women with autistic traits. Autism. (2020) 24:809–21. doi: 10.1177/1362361320912147 32429817

[44] Cook J Hull L Crane L Mandy W . Camouflaging in autism: A systematic review. Clin Psychol Rev. (2021) 89:102080. doi: 10.31219/osf.io/u5b9e 34563942

[45] Hull L Levy L Lai M-C Petrides KV Baron-Cohen S Allison C . Is social camouflaging associated with anxiety and depression in autistic adults? Mol Autism. (2021) 12:13. doi: 10.1186/s13229-021-00421-1 33593423 PMC7885456

[46] Bouaddi O Affane A Al malki C Souidi Y Khalis M . Access to autism services and support needs in Morocco: Perspectives of autistic adults, caregivers, service providers, and civil society. Children Youth Serv Rev. (2025) 178:108547. doi: 10.1016/j.childyouth.2025.10854742574925

[47] Crompton CJ Hallett S Axbey H McAuliffe C Cebula K . ‘Someone like-minded in a big place’: Autistic young adults’ attitudes towards autistic peer support in mainstream education. Autism. (2023) 27:76–91. doi: 10.1177/13623613221081189 PMC9806484 35249357

[48] Riebel M Bureau R Rohmer O Clément C Weiner L . Self-compassion as an antidote to self-stigma and shame in autistic adults. Autism. (2025) 29:1569–84. doi: 10.1177/13623613251316965 39959965

[49] Scior K Cooper R Fenn K Poole L Colman S Ali A . ‘Standing up for myself’ (STORM): Development and qualitative evaluation of a psychosocial group intervention designed to increase the capacity of people with intellectual disabilities to manage and resist stigma. J Appl Res Intellectual Disabil. (2022) 35:1297–306. doi: 10.1111/jar.13018 35785506 PMC9795919

[50] Alhusayni A Sheppard E Mitchell P Marsh L . How do autistic students do in the eyes of their peers? Non-autistic judgments about the academic experiences of autistic and non-autistic university students, based on brief samples of behavior. Autism Adulthood. (2025) 7:786–92. doi: 10.1089/aut.2023.0049 41625305 PMC12851862

[51] Treweek C Wood C Martin J Freeth M . Autistic people’s perspectives on stereotypes: An interpretative phenomenological analysis. Autism. (2019) 23:759–69. doi: 10.1177/1362361318778286 29848001

[52] Soeker MS . A descriptive, qualitative study of the challenges that individuals with Autism Spectrum Disorder experience when transitioning from skills training programs into the open labor market in Cape Town, South Africa. Work. (2020) 65:733–47. doi: 10.3233/wor-203127 32310205

[53] Baldwin S Costley D . The experiences and needs of female adults with high-functioning autism spectrum disorder. Autism. (2016) 20:483–95. doi: 10.1177/1362361315590805 26111537

[54] Huang Y Hwang YI Arnold SRC Lawson LP Richdale AL Trollor JN . Autistic adults’ experiences of diagnosis disclosure. J Autism Dev Disord. (2022) 52:5301–7. doi: 10.1007/s10803-021-05384-z 34978025

[55] Marion A Bowman K Thomas G Harrison AJ . A mixed method examination: how stigma experienced by autistic adults relates to metrics of social identity and social functioning. Front Psychiatry. (2023) 14:2023. doi: 10.3389/fpsyt.2023.1243618 37965369 PMC10640997

[56] Cage E Di Monaco J Newell V . Understanding, attitudes and dehumanisation towards autistic people. Autism. (2019) 23:1373–83. doi: 10.1177/1362361318811290 30463431

[57] Fukushima Y Yajima Y Moriyama M . Stigmatizing attitudes toward autistic students: A cross-sectional vignette survey among Japanese university students. Japan J Nurs Sci. (2025) 22:e70008. doi: 10.1111/jjns.70008 40152205

[58] Whelpley CE May CP . Seeing is disliking: Evidence of bias against individuals with autism spectrum disorder in traditional job interviews. J Autism Dev Disord. (2023) 53:1363–74. doi: 10.1007/s10803-022-05432-2 35294714

[59] Kim SY Song D-Y Bottema-Beutel K Gillespie-Lynch K . Time to level up: A systematic review of interventions aiming to reduce stigma toward autistic people. Autism. (2024) 28:798–815. doi: 10.1177/13623613231205915 37886792

[60] Massa A DeNigris D Gillespie-Lynch K . Theatre as a tool to reduce autism stigma? evaluating ‘Beyond Spectrums'. Res Drama Education: J Appl Theatre Perform. (2020) 25:613–30. doi: 10.1080/13569783.2020.173281037339054

[61] Gillions A O’Nions E Mansour H Hoare S Mandy W Stott J . The healthcare experiences of middle and older age autistic women in the United Kingdom. Autism. (2026) 30:49–60. doi: 10.1177/13623613251362265 40792546 PMC12717282

[62] Grahame C Thompson AR Freeth M . A wild and inaccessible place”: The lived experiences of autistic pregnancy and birth. Autism Adulthood. doi: 10.1089/aut.2024.003336698420

[63] Nicolaidis C Raymaker DM Ashkenazy E McDonald KE Dern S Baggs AE . Respect the way I need to communicate with you”: Healthcare experiences of adults on the autism spectrum. Autism. (2015) 19:824–31. doi: 10.1177/1362361315576221 25882392 PMC4841263

[64] Stein Duker LI Goodman E Pomponio Davidson A Mosqueda L . Caregiver perspectives on barriers and facilitators to primary care for autistic adults: A qualitative study. Front Med. (2022) 9:2022. doi: 10.3389/fmed.2022.1022026 36438029 PMC9685801

[65] Harada N Pellicano E Kumagaya S Ayaya S Asada K Senju A . ‘I don’t think they understand the reality of autism’: The lived experiences of autistic adults in Japan. Autism. (2025) 29:2715–26. doi: 10.1177/13623613251355303 PMC12531388 40684356

[66] Yoon W Seo J Je C . Korean autistic persons facing systemic stigmatization from middle education schools: daily survival on the edge as a puppet. Front Psychiatry. (2024) 15:2024. doi: 10.3389/fpsyt.2024.1260318 38606409 PMC11007699

[67] Gargallo-Nieto L Rodríguez-Zafra A Clemente-Sánchez M Garcia-Molina I . Late diagnosis of autistic women in Spain: A participatory study. Women's Stud Int Forum. (2025) 113:103177. doi: 10.1016/j.wsif.2025.10317742574925

[68] Mason D Ingham B Urbanowicz A Michael C Birtles H Woodbury-Smith M . A systematic review of what barriers and facilitators prevent and enable physical healthcare services access for autistic adults. J Autism Dev Disord. (2019) 49:3387–400. doi: 10.1007/s10803-019-04049-2 31124030 PMC6647496

[69] Romualdez AM Walker Z Remington A . Autistic adults' experiences of diagnostic disclosure in the workplace: Decision-making and factors associated with outcomes. Autism Dev Lang Impair. (2021) 6:23969415211022955. doi: 10.1177/23969415211022955 36381532 PMC9620671

[70] Marsack CN Perry TE . Aging in place in every community: Social exclusion experiences of parents of adult children with autism spectrum disorder. Res Aging. (2018) 40:535–57. doi: 10.1177/0164027517717044 28677419 PMC6345512

[71] Milačić-Vidojević I Gligorović M Dragojević N . Tendency towards stigmatization of families of a person with autistic spectrum disorders. Int J Soc Psychiatry. (2014) 60:63–70. doi: 10.1177/0020764012463298 23117824

[72] Oti-Boadi M Oppong Asante K Malm EK . The experiences of ageing parents of young adults with autism spectrum disorders (ASD). J Adult Dev. (2020) 27:58–69. doi: 10.1007/s10804-018-09325-630311153

[73] Clarke EB McCauley JB Lutz A Gotelli M Sheinkopf SJ Lord C . Understanding profound autism: Implications for stigma and supports. Front Psychiatry. (2024) 15:2024. doi: 10.3389/fpsyt.2024.1287096 38317766 PMC10839016

[74] Lodder A Papadopoulos C Randhawa G . SOLACE: A psychosocial stigma protection intervention to improve the mental health of parents of autistic children-a feasibility randomised controlled trial. J Autism Dev Disord. (2020) 50:4477–91. doi: 10.1007/s10803-020-04498-0 32323046 PMC7677275

[75] Bowleg L . The problem with the phrase women and minorities: Intersectionality-an important theoretical framework for public health. Am J Public Health. (2012) 102:1267–73. doi: 10.2105/ajph.2012.300750 22594719 PMC3477987

[76] Doyle N McDowall A Waseem U . Intersectional stigma for autistic people at work: A compound adverse impact effect on labor force participation and experiences of belonging. Autism Adulthood. (2022) 4:340–56. doi: 10.1089/aut.2021.0082 36777372 PMC9908290

[77] Crenshaw K . Demarginalizing the intersection of race and sex: A black feminist critique of antidiscrimination doctrine, feminist theory and antiracist policies. Univ Chicago Legal Forum. (1989) 1989:139–67. doi: 10.4324/9780429499142-5

[78] Crenshaw K . Mapping the margins: Intersectionality, identity politics, and violence against women of color. Stanford Law Rev. (1991) 43:1241–99. doi: 10.2307/1229039

[79] Pyszkowska A Nowacki A Stefanek F . Loud and proud: Collective self-esteem is negatively associated with internalized stigma and minority stress. A mixed methods study of intersectional stigma among queer autistic adults from Poland. Res Autism. (2026) 130:202781. doi: 10.1016/j.reia.2025.20278142574925

[80] Wallisch A Boyd BA Hall JP Kurth NK Streed CG Mulcahy A . Health care disparities among autistic LGBTQ+ people. Autism Adulthood. (2023) 5:165–74. doi: 10.1089/aut.2022.0006 37346996 PMC10280185

[81] Logie CH Earnshaw V Nyblade L Turan J Stangl A Poteat T . A scoping review of the integration of empowerment-based perspectives in quantitative intersectional stigma research. Glob Public Health. (2022) 17:1451–66. doi: 10.1080/17441692.2021.1934061 34061710

[82] Bachmann CJ Höfer J Kamp-Becker I Küpper C Poustka L Roepke S . Internalised stigma in adults with autism: A German multi-center survey. Psychiatry Res. (2019) 276:94–9. doi: 10.1016/j.psychres.2019.04.023 31030006

[83] Bakombo S Ewalefo P Konkle ATM . The influence of social media on the perception of autism spectrum disorders: Content analysis of public discourse on YouTube videos. Int J Environ Res Public Health. (2023) 20:3246. doi: 10.3390/ijerph20043246 36833941 PMC9961260

[84] Brosnan M Gavin J . The impact of stigma, autism label and wording on the perceived desirability of the online dating profiles of men on the autism spectrum. J Autism Dev Disord. (2021) 51:4077–85. doi: 10.1007/s10803-020-04830-8 33459917 PMC8510896

[85] Bury SM Haschek A Wenzel M Spoor JR Hedley D . Brief report: Learning about autism: Is the source of autism knowledge associated with differences in autism knowledge, autism identity, and experiences of stigma. J Autism Dev Disord. (2024) 54:4346–53. doi: 10.1007/s10803-022-05823-5 36443495

[86] Caron V Jeanneret N Giroux M Guerrero L Ouimet M Forgeot d’Arc B . Sociocultural context and autistics’ quality of life: A comparison between Québec and France. Autism. (2022) 26:900–13. doi: 10.1007/978-1-349-06324-6_2 34325546 PMC9008549

[87] Cheng Y Dwyer P Keating CT . Factors underlying differences in knowledge, explicit stigma and implicit biases towards autism across Hong Kong, the United Kingdom and the United States. Autism. (2025) 29:868–83. doi: 10.1177/13623613241290565 39487729 PMC11967188

[88] Dambrun M Marinho G Mondillon L Clinchamps M Dutheil F Chakroun N . Social perceptions and the stigmatization towards fifteen mental illnesses in France: A preliminary study on the role of vital force and burden. Front Psychiatry. (2024) 15:2024. doi: 10.3389/fpsyt.2024.1336690 38550539 PMC10973838

[89] Davis AM Smith E Yang X Wright R . Exploring racial discrimination, disability discrimination, and perception of the future among black-identifying emerging adults with and without autism in the United States: A mixed-methods descriptive study. J Child Adolesc Trauma. (2024) 17:1019–34. doi: 10.1007/s40653-024-00624-7 39686937 PMC11646248

[90] Liu F Begeer S Hoekstra RA Wang C Scheeren AM . Self-reported psychosocial outcomes of autistic adults living in China and the Netherlands. Autism Adulthood. doi: 10.1089/aut.2024.0073 38864577

[91] McLeod JD Meanwell E Hawbaker A . The experiences of college students on the autism spectrum: A comparison to their neurotypical peers. J Autism Dev Disord. (2019) 49:2320–36. doi: 10.1007/s10803-019-03910-8 30734173

[92] Nimante D Laganovska E Osgood R . To tell or not to tell – disclosure of autism in the workplace. Int J Dev Disabil. (2023) 69:414–23. doi: 10.1080/20473869.2023.2193489 37213588 PMC10197981

[93] Soffer M Argaman-Danos S . Self-labeling, perceived stigma toward autism spectrum disorder, and self-esteem and the change in autism nosology. Disability Health J. (2021) 14:101162. doi: 10.1016/j.dhjo.2021.101162 34229978

[94] Tan DW Rabuka M Haar T Pellicano E . ‘It’s a symbolic violence’: Autistic people’s experiences of discrimination at universities in Australia. Autism. (2024) 28:1344–56. doi: 10.1177/13623613231219744 PMC11134995 38148639

[95] Ward N Randle-Phillips C Aldridge R . Autistic adults and loved ones’ experiences of a later life diagnosis. J Autism Dev Disord. (2026). doi: 10.1007/s10803-026-07232-4 41639542

