# Supplementary material for: Quality of life outcomes associated with stigma in autistic adults and their families: a systematic review
Source: Front Psychiatry. 2026 Sep 30;17:1911423. doi: 10.3389/fpsyt.2026.1911423 (PMC13662678; doi:10.3389/fpsyt.2026.1911423)
Supplement: Supplementary file 1 [file Table1.docx]

Supplementary Material

# 1 Full Search Strategy

## 1.1 APA PsycInfo <1806 to February 2026 Week 2>

| **#** | **Query** | **Results from 17 Feb 2026** |
| --- | --- | --- |
| 1 | exp autism spectrum disorders/ or exp autistic traits/ or exp "autism spectrum disorders (attitudes toward)"/ or exp neurodiversity/ | 66,274 |
| 2 | autis*.mp. [mp=title, abstract, heading word, table of contents, key concepts, original title, tests & measures, mesh word] | 81,299 |
| 3 | Asperger*.mp. [mp=title, abstract, heading word, table of contents, key concepts, original title, tests & measures, mesh word] | 4,849 |
| 4 | pervasive developmental disorder*.mp. [mp=title, abstract, heading word, table of contents, key concepts, original title, tests & measures, mesh word] | 3,349 |
| 5 | autism spectrum.mp. [mp=title, abstract, heading word, table of contents, key concepts, original title, tests & measures, mesh word] | 70,563 |
| 6 | 1 or 2 or 3 or 4 or 5 | 82,329 |
| 7 | exp stigma/ or exp mental health stigma/ or exp self-stigma/ | 23,486 |
| 8 | stigma*.mp. [mp=title, abstract, heading word, table of contents, key concepts, original title, tests & measures, mesh word] | 56,153 |
| 9 | discriminat*.mp. [mp=title, abstract, heading word, table of contents, key concepts, original title, tests & measures, mesh word] | 181,951 |
| 10 | prejudice*.mp. [mp=title, abstract, heading word, table of contents, key concepts, original title, tests & measures, mesh word] | 27,821 |
| 11 | stereotype*.mp. [mp=title, abstract, heading word, table of contents, key concepts, original title, tests & measures, mesh word] | 46,213 |
| 12 | negative attitude*.mp. [mp=title, abstract, heading word, table of contents, key concepts, original title, tests & measures, mesh word] | 10,472 |
| 13 | inequalit*.mp. [mp=title, abstract, heading word, table of contents, key concepts, original title, tests & measures, mesh word] | 37,744 |
| 14 | social exclusion.mp. or exp Social Exclusion/ | 5,304 |
| 15 | 7 or 8 or 9 or 10 or 11 or 12 or 13 or 14 | 329,578 |
| 16 | exp Middle Adulthood/ or exp Emerging Adulthood/ or exp Older Adulthood/ | 45,338 |
| 17 | adult*.mp. [mp=title, abstract, heading word, table of contents, key concepts, original title, tests & measures, mesh word] | 1,154,047 |
| 18 | 16 or 17 | 1,154,047 |
| 19 | 6 and 15 and 18 | 1,458 |
| 20 | limit 19 to (human and english language and yr="2013 -Current") | 788 |

## 1.2 Embase Classic+Embase <1947 to 2026 February 13>

| **#** | **Query** | **Results from 17 Feb 2026** |
| --- | --- | --- |
| 1 | exp autism/ or exp asperger syndrome/ or exp "pervasive developmental disorder not otherwise specified"/ | 124,573 |
| 2 | autis*.mp. [mp=title, abstract, heading word, drug trade name, original title, device manufacturer, drug manufacturer, device trade name, keyword heading word, floating subheading word, candidate term word] | 132,914 |
| 3 | Asperger*.mp. [mp=title, abstract, heading word, drug trade name, original title, device manufacturer, drug manufacturer, device trade name, keyword heading word, floating subheading word, candidate term word] | 6,815 |
| 4 | Pervasive Developmental Disorder*.mp. [mp=title, abstract, heading word, drug trade name, original title, device manufacturer, drug manufacturer, device trade name, keyword heading word, floating subheading word, candidate term word] | 4,226 |
| 5 | autism spectrum.mp. [mp=title, abstract, heading word, drug trade name, original title, device manufacturer, drug manufacturer, device trade name, keyword heading word, floating subheading word, candidate term word] | 64,312 |
| 6 | 1 or 2 or 3 or 4 or 5 | 140,658 |
| 7 | exp social stigma/ or exp stigma/ or exp self stigma/ | 51,826 |
| 8 | stigma*.mp. [mp=title, abstract, heading word, drug trade name, original title, device manufacturer, drug manufacturer, device trade name, keyword heading word, floating subheading word, candidate term word] | 106,407 |
| 9 | discriminat*.mp. [mp=title, abstract, heading word, drug trade name, original title, device manufacturer, drug manufacturer, device trade name, keyword heading word, floating subheading word, candidate term word] | 494,936 |
| 10 | prejudice*.mp. [mp=title, abstract, heading word, drug trade name, original title, device manufacturer, drug manufacturer, device trade name, keyword heading word, floating subheading word, candidate term word] | 13,889 |
| 11 | stereotype*.mp. [mp=title, abstract, heading word, drug trade name, original title, device manufacturer, drug manufacturer, device trade name, keyword heading word, floating subheading word, candidate term word] | 27,203 |
| 12 | negative attitude*.mp. [mp=title, abstract, heading word, drug trade name, original title, device manufacturer, drug manufacturer, device trade name, keyword heading word, floating subheading word, candidate term word] | 11,981 |
| 13 | inequalit*.mp. [mp=title, abstract, heading word, drug trade name, original title, device manufacturer, drug manufacturer, device trade name, keyword heading word, floating subheading word, candidate term word] | 83,343 |
| 14 | exp social exclusion/ or social exclusion.mp. | 6,405 |
| 15 | 7 or 8 or 9 or 10 or 11 or 12 or 13 or 14 | 713,693 |
| 16 | exp young adult/ or exp adult/ | 13,691,562 |
| 17 | adult*.mp. [mp=title, abstract, heading word, drug trade name, original title, device manufacturer, drug manufacturer, device trade name, keyword heading word, floating subheading word, candidate term word] | 12,744,711 |
| 18 | 16 or 17 | 14,482,762 |
| 19 | 6 and 15 and 18 | 2,743 |
| 20 | limit 19 to (human and english language and yr="2013 -Current") | 2,021 |

## 1.3 Ovid MEDLINE(R) ALL <1946 to February 13, 2026>

| **#** | **Query** | **Results from 17 Feb 2026** |
| --- | --- | --- |
| 1 | exp child development disorders, pervasive/ or exp autism spectrum disorder/ or exp asperger syndrome/ or exp autistic disorder/ | 56,501 |
| 2 | autis*.mp. [mp=title, book title, abstract, original title, name of substance word, subject heading word, floating sub-heading word, keyword heading word, organism supplementary concept word, protocol supplementary concept word, rare disease supplementary concept word, unique identifier, synonyms, population supplementary concept word, anatomy supplementary concept word] | 87,221 |
| 3 | Asperger*.mp. [mp=title, book title, abstract, original title, name of substance word, subject heading word, floating sub-heading word, keyword heading word, organism supplementary concept word, protocol supplementary concept word, rare disease supplementary concept word, unique identifier, synonyms, population supplementary concept word, anatomy supplementary concept word] | 3,010 |
| 4 | pervasive developmental disorder*.mp. [mp=title, book title, abstract, original title, name of substance word, subject heading word, floating sub-heading word, keyword heading word, organism supplementary concept word, protocol supplementary concept word, rare disease supplementary concept word, unique identifier, synonyms, population supplementary concept word, anatomy supplementary concept word] | 2,186 |
| 5 | autism spectrum.mp. [mp=title, book title, abstract, original title, name of substance word, subject heading word, floating sub-heading word, keyword heading word, organism supplementary concept word, protocol supplementary concept word, rare disease supplementary concept word, unique identifier, synonyms, population supplementary concept word, anatomy supplementary concept word] | 52,060 |
| 6 | exp Social Stigma/ | 17,333 |
| 7 | stigma*.mp. | 73,141 |
| 8 | discriminat*.mp. [mp=title, book title, abstract, original title, name of substance word, subject heading word, floating sub-heading word, keyword heading word, organism supplementary concept word, protocol supplementary concept word, rare disease supplementary concept word, unique identifier, synonyms, population supplementary concept word, anatomy supplementary concept word] | 372,094 |
| 9 | prejudice*.mp. [mp=title, book title, abstract, original title, name of substance word, subject heading word, floating sub-heading word, keyword heading word, organism supplementary concept word, protocol supplementary concept word, rare disease supplementary concept word, unique identifier, synonyms, population supplementary concept word, anatomy supplementary concept word] | 32,454 |
| 10 | stereotype*.mp. [mp=title, book title, abstract, original title, name of substance word, subject heading word, floating sub-heading word, keyword heading word, organism supplementary concept word, protocol supplementary concept word, rare disease supplementary concept word, unique identifier, synonyms, population supplementary concept word, anatomy supplementary concept word] | 28,031 |
| 11 | negative attitude*.mp. [mp=title, book title, abstract, original title, name of substance word, subject heading word, floating sub-heading word, keyword heading word, organism supplementary concept word, protocol supplementary concept word, rare disease supplementary concept word, unique identifier, synonyms, population supplementary concept word, anatomy supplementary concept word] | 9,767 |
| 12 | inequalit*.mp. [mp=title, book title, abstract, original title, name of substance word, subject heading word, floating sub-heading word, keyword heading word, organism supplementary concept word, protocol supplementary concept word, rare disease supplementary concept word, unique identifier, synonyms, population supplementary concept word, anatomy supplementary concept word] | 65,366 |
| 13 | social exclusion.mp. [mp=title, book title, abstract, original title, name of substance word, subject heading word, floating sub-heading word, keyword heading word, organism supplementary concept word, protocol supplementary concept word, rare disease supplementary concept word, unique identifier, synonyms, population supplementary concept word, anatomy supplementary concept word] | 3,356 |
| 14 | 1 or 2 or 3 or 4 or 5 | 89,143 |
| 15 | 6 or 7 or 8 or 9 or 10 or 11 or 12 or 13 | 554,040 |
| 16 | exp Adult/ or exp Young Adult/ | 8,681,773 |
| 17 | adult*.mp. [mp=title, book title, abstract, original title, name of substance word, subject heading word, floating sub-heading word, keyword heading word, organism supplementary concept word, protocol supplementary concept word, rare disease supplementary concept word, unique identifier, synonyms, population supplementary concept word, anatomy supplementary concept word] | 7,182,633 |
| 18 | 16 or 17 | 9,563,674 |
| 19 | 14 and 15 and 18 | 1,622 |
| 20 | limit 19 to (english language and humans and yr="2013 -Current") | 847 |
